# P01.13. Assessing cognitive decline and cardiac senescence in middle aged and elderly volunteers: implications for herbal medicine utilization

**DOI:** 10.1186/1472-6882-12-S1-P13

**Published:** 2012-06-12

**Authors:** D Oh, S Park, C Jo, H Yoo

**Affiliations:** 1 Cancer Research Center, Korea Inst. of Oriental Med. (KIOM), Daejeon, Republic of Korea; 2 Internal Med., Dunsan Oriental Medical Hospital of Daejeon Univ., Daejeon, Republic of Korea

## Purpose

Although the aging process appears to be generally characterized, much attention has been paid to the specific target molecules to discover clinical surrogate markers of senescence. The present study is to investigate the phenotypic profile of cognitive decline and functional cardiac senescence in healthy middle aged and elderly volunteers.

## Methods

This study was conducted as an open, cross-sectional, single-center, comparative clinical study. The IRB-approved protocol of this study is in the process of being registered with the NIH (clinicaltrials.gov). The sample size was a total of 72 participants who had no past illness or had taken any concomitant medications. Participants were divided into two groups of middle aged (45-64) and elderly (over 65) participants. Each volunteer was given informed consent for checking cognition (Modified MMSE, ADAS-cog) and cardiac function (HRV). Blood and urine samples were collected to analyze genomic, proteomic, and metabolomic markers related to cognition (APOε4, type-3 metabotropic glutamate R) and cardiac function (CRP, cardiac troponin T).

## Results

No demographic differences were observed between the two groups (p = 0.336). There was a significant difference between the two groups in terms of ADAS-cog total score (3.3 points, p = 0.002) and modified-MMSE, immediate and delayed recall, by 1 point (p = 0.047).

## Conclusion

This study characterized the phenotypic difference of ADAS-cog and modified MMSE testing between healthy middle aged and elderly participants. The molecular findings from genomic, proteomic, and metabolomic analyses provide implication for herbal medicine and its application in modulating multi-target molecules which affect the senescence of specific organs. This study also provides implications for healthy elderly people as a historical control group for conducting studies enrolling patients with Alzheimer’s disease.

